# The Forgotten Triangular Space: Anatomy, Disease Mechanisms, and Contemporary Management of Interdental Embrasures—A Narrative Review

**DOI:** 10.7759/cureus.102114

**Published:** 2026-01-22

**Authors:** Shweta Ann Jacob, Reshma Suresh, Shankar S Menon, Arun Kurumathur Vasudevan, Biju Balakrishnan, Maya Rajan Peter

**Affiliations:** 1 Periodontics, Amrita School of Dentistry, Amrita Vishwa Vidyapeetham, Kochi, IND

**Keywords:** black triangles, connective tissue graft, embrasure space, gingival biotype, hyaluronic acid, interdental papilla, papilla reconstruction, periodontal aesthetics

## Abstract

The interdental papilla forms a critical component of the gingival complex, occupying the embrasure space between adjacent teeth and contributing to aesthetic harmony, phonetic integrity, and defense against food impaction and microbial colonization. Loss of papillary height, manifesting as open embrasures or "black triangles," arises from diverse etiologies, including periodontal disease progression, traumatic hygiene practices, divergent root angulations, aging-related tissue atrophy, and iatrogenic factors. This condition not only compromises smile aesthetics but also predisposes to plaque retention, root sensitivity, and localized inflammation. With increasing patient awareness of cosmetic outcomes, effective maintenance and reconstruction of embrasure spaces have gained prominence in periodontal therapy.

## Introduction and background

Pristine periodontal health represents an idealized state characterized by the complete absence of clinical inflammation and preserved supporting structures, including intact attachment levels and alveolar bone [1]. Clinically, such conditions are rarely encountered, as subtle inflammatory changes frequently coexist with physiological immune surveillance [1]. The American Academy of Periodontology defines health functionally as the absence of disease or abnormality, encompassing gingival tissues free from destructive processes [2]. Gingival inflammation, initiated by biofilm accumulation, manifests as site-specific redness and edema without attachment loss, often evading patient detection due to its painless and insidious nature [3].

Periodontitis, conversely, constitutes a chronic multifactorial disease driven by dysbiotic plaque, resulting in progressive destruction of the tooth-supporting apparatus. Hallmarks include clinical attachment loss, radiographic bone resorption, pocket formation, and gingival bleeding [4]. This condition exerts a profound public health impact, contributing to edentulism, masticatory dysfunction, aesthetic compromise, and elevated care costs, with plausible systemic implications [4]. Global burden analyses reveal an 8.44% rise in age-standardized prevalence of severe periodontitis from 1990 to 2019, disproportionately affecting developing regions, where population growth accounted for 67.9% of new cases [5].

Amid escalating cosmetic demands, gingival aesthetics has emerged as a focal concern, particularly in interdental regions. The embrasure space, the triangular area bounded by contact points, alveolar crest, and gingival contours, plays a pivotal role in smile design [6]. Its complex anatomy and vascular supply underpin both health maintenance and appearance. The gingiva comprises free, attached, and interdental components. Free gingiva forms a 1-mm collar demarcated by the gingival groove in 50% of cases, enclosing the sulcus with probing depths of 2-3 mm in health [7]. Attached gingiva, keratinized and periosteum-bound, varies regionally (3.5-4.5 mm maxillary incisors; 3.3-3.9 mm mandibular), widening with age and supra-eruption [7].

Interdental gingiva, or papilla, occupies embrasures, assuming narrower pyramidal forms anteriorly due to reduced tooth mass and broader, col-shaped configurations posteriorly [8]. Cohen's morphological description highlights the "col" as a concave depression facilitating self-cleansing [8]. Factors modulating shape include interproximal bone height, root proximity, crown form, and biotype [9]. Tarnow's criterion establishes that papilla fill is nearly universal at ≤5 mm bone-to-contact distance, dropping to 27% at 7 mm [9]. Thin, scalloped biotypes predispose to recession, contrasting thick, flat variants [10].

Developmentally, children's papillae appear bulbous, maturing with eruption [6]. In adults, aging induces epithelial thinning and height reduction (approximately 0.012 mm/year) [6]. The papilla's functions extend beyond aesthetics to preventing food lodgment, phonetic modulation, and plaque deflection [11]. Frenum anomalies contribute to midline diastemas by impeding closure, exacerbating embrasure widening [12]. Food impaction, classified vertically or horizontally, arises from open contacts or plunger cusps, fostering inflammation [13].

Pathogenesis of papillary loss involves inflammatory cascades degrading junctional epithelium and connective tissue, compounded by traumatic hygiene, divergent roots, and iatrogenic restorations [14]. "Black triangles" signify recession, impairing aesthetics and retention [15]. The absence of a periodontal ligament in dental implants, unlike natural teeth, creates flatter gingiva between implants, elevating recession risk [16]. Classifications such as Nordland and Tarnow's help to grade recession by cemento-enamel junction reference, guiding prognosis [17]. It is therefore imperative that black triangles are resolved so as to aid in aesthetics.

Management paradigms span non-surgical and surgical domains. Non-surgical approaches correct etiology via orthodontics, injectables, or restoratives; surgical methods reconstruct via grafting and flap mobilization [18-20]. This review integrates anatomical, pathophysiological, and therapeutic insights to optimize embrasure maintenance in contemporary practice.

## Review

Methodology

This review was conducted as a narrative synthesis aimed at integrating anatomical, developmental, pathophysiological, and clinical evidence related to interdental papilla loss and its management. The primary objective was to provide a biologically informed and clinically oriented overview of existing classification systems and therapeutic strategies rather than to perform quantitative effect-size comparisons or meta-analytic pooling. Accordingly, the review emphasizes conceptual coherence, clinical relevance, and mechanistic plausibility across heterogeneous sources of evidence.

A targeted literature search was performed using PubMed/MEDLINE, Scopus, and Google Scholar to identify relevant publications from database inception through October 31, 2025. Search strategies were adapted to each database and employed combinations of keywords and Boolean operators, including terms related to interdental papilla anatomy, papilla loss, embrasure space, black triangles, gingival biotype, hyaluronic acid-based therapies, platelet-rich fibrin (PRF), connective tissue grafting, papilla preservation flaps, and orthodontic approximation. Reference lists of key reviews, consensus documents, and seminal articles were also manually screened to ensure comprehensive coverage of foundational and influential studies.

Eligibility criteria included human studies addressing interdental papilla anatomy, development, classification, etiopathogenesis, or clinical management. Randomized controlled trials, cohort and case-control studies, case series, and case reports describing surgical techniques, systematic or narrative reviews, and consensus statements were considered eligible. Textbook sources, including Carranza’s Clinical Periodontology, were used selectively to support anatomical and histological descriptions. Studies were excluded if they were limited to animal or in vitro models without clear translational relevance, lacked full-text availability, were not published in English, or represented opinion-based commentary without empirical or methodological support.

Study selection was performed through sequential screening of titles, abstracts, and full texts based on relevance to the review objectives. Given the narrative design of the review, formal dual independent screening was not conducted; however, study inclusion followed predefined eligibility criteria and thematic relevance. Data extraction focused on study design, level of evidence, anatomical or histological findings, classification frameworks, reported clinical outcomes related to papillary height and embrasure fill, and descriptions of surgical or non-surgical management strategies.

Evidence synthesis was qualitative and thematic. Management approaches were grouped into non-surgical and surgical modalities, and their rationale was interpreted in the context of vascular preservation, scaffold stability, soft tissue thickness, and tension-free wound closure. Quantitative values reported in the literature, such as papillary height gain or percentage embrasure fill, were summarized descriptively as ranges observed across individual studies and were not statistically pooled or weighted.

A formal risk-of-bias assessment was not undertaken, as the review did not aim to compare interventions quantitatively or generate pooled effect estimates. Many included studies consisted of case series or technique descriptions, for which formal bias scoring would not meaningfully enhance interpretation within a narrative framework. Instead, the strength of evidence was appraised qualitatively based on study design hierarchy, consistency of findings, and biological plausibility supported by histological and clinical data. Given the substantial heterogeneity in defect morphology, outcome definitions, measurement techniques, and follow-up intervals across studies, a narrative synthesis was considered the most appropriate and clinically meaningful approach for addressing the objectives of this review.

Results

*Anatomical Foundations* 

Gingival tissues exhibit specialized architecture for barrier function. Macroscopically, free gingiva (1 mm wide) forms the sulcus wall, attached gingiva (keratinized, 3-4 mm) anchors to bone, and interdental papilla fills embrasures [7]. Microscopically, orthokeratinized epithelium overlays dense collagen, with rete pegs enhancing adhesion [7]. Vascular supply via supraperiosteal and periodontal ligament vessels sustains papilla viability [6].

Interdental papilla shape varies, it appears pyramidal in anterior regions (with smaller tissue mass) and col-depressed in posterior regions [8]. The location of the contact point dictates papilla height; a 5 mm distance from bone ensures complete fill [9]. Phenotype influences outcomes: thin phenotypes are scalloped and translucent, whereas thick phenotypes are flat and opaque.

*Developmental and Age-Related Variations* 

Pediatric papillae are voluminous, reflecting eruption dynamics; adult forms stabilize after maturation [6]. Aging reduces height via epithelial attenuation and collagen degradation [6].

*Functional Roles* 

The interdental papilla serves multiple functional roles beyond aesthetics: it prevents food impaction, facilitates phonetic articulation, and deflects plaque from interproximal areas [11]. Aberrant frenum can perpetuate midline diastemas, thereby widening embrasure spaces [12]. Proper classification of food lodgment (vertical or horizontal) guides restorative interventions to eliminate interproximal plaque traps [13].

*Pathophysiology and Classification* 

Papillary loss initiates with biofilm-induced inflammation, progressing to attachment migration and bone resorption [14]. Black triangles correlate with >5 mm bone-contact distance [15]. The Nordland-Tarnow classification assesses interdental papilla loss based on cementoenamel junction exposure [17]. Implant gingiva lacks ligament support, forming flatter contours [16].

*Management Approaches* 

Non-surgical approaches encompass several minimally invasive strategies.

Hyaluronic acid: Hyaluronic acid injections, administered in doses of 0.1-0.2 mL with repeated applications as needed, enhance tissue volume and produce height gains ranging from 0.2 to 0.85 mm, achieving 50%-90% embrasure fill by the six-month mark [20].

Platelet concentrates: Recent randomized split‑mouth trials comparing hyaluronic acid and injectable platelet concentrates in thin gingival phenotypes provide direct clinical data on short‑term volumetric gains and patient‑reported outcomes, informing choice between these minimally invasive options [18,19]. PRF delivers concentrated growth factors that promote rapid tissue regeneration, resulting in 90%-100% short-term fill of the papillary defect [21-23].

Orthodontic approximation: Orthodontic intervention effectively closes interdental spaces, yielding sustainable height increases of 1-3 mm through controlled tooth approximation [22]. Restorative contouring camouflages black triangles by strategically modifying crown morphology to optically reduce visible gaps [23].

Surgical interventions target more pronounced defects with reconstructive precision.

Connective tissue grafts: Connective tissue grafts, harvested and positioned via envelope flaps, restore 1-3 mm of papillary height and provide 80%-100% coverage of the embrasure area [24]. Semilunar incisions, as described by Han and Takei, along with modified Beagle techniques, limit surgical access to reduce trauma, decreasing black triangle dimensions by 40%-70% [25]. Tube-based methods create contained pouches for graft placement, making them particularly suitable for managing wide embrasure defects [26].

Maintenance protocols center on daily interdental hygiene using soft brushes, floss threaders, and periodic professional recalls to sustain long-term stability [6].

Data Extraction and Synthesis

Data were extracted independently from the included studies focusing on interdental papilla reconstruction techniques. Extracted variables included study design, level of evidence, reported mean papillary height gain, percentage of embrasure fill at defined follow-up intervals, longevity of outcomes, and qualitative assessments of predictability. Owing to substantial clinical and methodological heterogeneity across studies, including differences in papilla classification systems, defect morphology, surgical protocols, adjunctive materials, outcome measurement techniques, and follow-up durations, formal quantitative synthesis (meta-analysis or meta-regression) was not feasible.

Accordingly, Table 1 presents a descriptive synthesis in which numerical values represent ranges or typical results reported in individual studies, rather than pooled or weighted estimates. No fixed- or random-effects models were applied, and no heterogeneity metrics, confidence intervals, or hypothesis testing were calculated.

**Table 1:** Shows a comparative overview of interdental papilla reconstruction techniques This table provides a descriptive narrative summary of outcomes reported in the included studies for various interdental papilla reconstruction techniques. Reported values for mean papillary height gain, percentage embrasure fill, longevity, and predictability reflect ranges or representative findings observed in individual studies. No formal statistical pooling, meta-analysis, or meta-regression was performed. Consequently, no weighted effect sizes, 95% confidence intervals, p-values, or heterogeneity estimates (e.g., I²) are presented. The data are intended to support qualitative clinical comparison rather than inferential statistical evaluation. RCT, randomized controlled trial. Table credits: Dr. Shweta Ann Jacob

Technique	Level of evidence	Mean papillary height gain	% embrasure fill (6–12 months)	Longevity	Predictability	Key reference
Hyaluronic acid injection	Systematic review + 2 RCTs	0.2–0.85 mm	50%–90% at 6 months	6–12 months (resorption)	Good for mild defects; requires repeats	[20]
Platelet-rich fibrin	Review + case series	0.5–1.2 mm (early)	90%–100% at 3 months	<6 months	Excellent short-term; fades without a scaffold	[21]
Orthodontic approximation	Cohort studies + case reports	1–3 mm	80%–90%	>2 years	Highly predictable when the etiology is divergence/diastema	[22]
Restorative contouring	Case series	Optical only (no true gain)	Immediate aesthetic gain	Permanent	Masking only, risk of plaque accumulation	[23]
Connective tissue graft (envelope/semilunar)	Multiple RCTs	1.5–3.5 mm	85%–100%	>24 months	Current gold standard: most predictable and stable	[24]
Han-Takei/modified Beagle	Case series	1–2.5 mm	40%–75%	12–24 months	Good in the anterior zone; minimally invasive	[25]
Tube technique	Case reports	2–4 mm (wide defects)	70%–90%	12–18 months	Useful for class III defects; technically demanding	[26]

Discussion

Embrasure space maintenance embodies the intersection of periodontal biology and aesthetic dentistry, demanding a nuanced understanding of anatomy and pathophysiology. The interdental papilla's vulnerability stems from its limited vascularity and dependence on underlying bone [9]. Tarnow's 5 mm threshold remains a prognostic cornerstone; distances exceeding this predictably yield black triangles, irrespective of intervention [9]. Phenotype modulates outcomes: Thin tissues, with delicate collagen and high scalloping, exhibit greater recession propensity and surgical complexity, often necessitating volumizing adjuncts [10]. Thick phenotypes, conversely, accommodate grafting robustly, supporting tension-free closure [10].

Pathogenesis underscores inflammation as the primary driver, with dysbiotic biofilms eroding junctional epithelium and triggering osteoclast activation [14]. Secondary factors, traumatic brushing, divergent roots, and plunger cusps, amplify loss through mechanical disruption [13]. Aging exacerbates via cumulative exposure and tissue remodeling, highlighting prevention in younger cohorts [6]. Frenum-mediated diastemas illustrate developmental persistence, resolvable orthodontically before papillary collapse [12].

Non-Surgical Techniques

Non-surgical modalities have transformed conservative management. Hyaluronic acid, a glycosaminoglycan, hydrates and stimulates fibroblasts, offering transient augmentation ideal for mild defects [20]. Its non-immunogenic profile and ease of delivery suit outpatient settings, though resorption mandates repetition [20]. Platelet concentrates such as PRF harness autologous cytokines for regeneration, outperforming controls in early healing but fading long-term without scaffolds [21]. Orthodontic approximation addresses etiology directly, migrating contacts coronally and inducing gingival creep-sustained results affirm its role in multidisciplinary protocols [22]. Restorative camouflage, via composite additions, provides immediate aesthetics but risks plaque if overcontoured [23].

Surgical Techniques

Surgical reconstruction remains the gold standard for advanced loss, where non-surgical reconstruction gains plateau. Subepithelial connective tissue grafts supply volume and vascularity, integrating seamlessly when harvested from the palate or tuberosity [24]. Flap designs critically influence success: envelope techniques preserve papillae, minimizing scarring; semilunar incisions (Han-Takei) enable coronal repositioning without vertical releases [25]. Modified Beagle approaches, incorporating buccal access, enhance precision in anterior zones [25]. Tube methods, creating pouches for graft containment, suit wide embrasures but demand microsurgical finesse [26].

A recent systematic review and meta‑analysis of injectable platelet‑rich fibrin for gingival phenotype modification synthesizes randomized data and clarifies short‑term volumetric effects and limitations in long‑term stability [27]. Complications such as necrosis and dehiscence arise from tension or vascular compromise, mitigated by tension-free closure and biomaterial adjuncts [24].

Implant contexts reveal biological disparities. Peri-implant mucosa forms parallel fibers without cementum insertion, yielding flatter papillae and >50% loss risk at >3 mm inter-implant distance [16]. Strategies employed here help to integrate platform switching, prosthetic emergence profiling, and soft tissue augmentation [16].

Maintenance protocols are paramount for longevity. Interdental aids such as brushes and floss threads prevent trauma, and powered devices enhance compliance [6]. Periodic reassessment detects early recession, enabling timely intervention. Patient education on biotype-specific hygiene tailors prevention.

A key limitation of this review is the absence of a formal quantitative synthesis. Although numerical ranges are presented to aid clinical interpretation, these values are descriptive in nature and do not represent pooled effect sizes or statistically comparable outcomes. The heterogeneity of study designs, outcome measures, and follow-up intervals precluded reliable meta-analytic integration.

Consequently, the findings should be interpreted as comparative trends rather than definitive estimates of treatment efficacy. Future investigations incorporating standardized outcome measures and adequately powered randomized controlled trials would permit robust meta-analytic evaluation, ideally conducted in collaboration with a biostatistician.

Future directions encompass biomaterials such as cross-linked hyaluronans for prolonged effect, stem cell-enriched matrices for true regeneration, and 3D-printed scaffolds for precision grafting. Digital planning via cone beam computed tomography-guided injections and intraoral scanning promises reproducibility [28]. Emerging regenerative approaches, including stem cell-based therapies and 3D bio-printed scaffolds for interdental papilla reconstruction, are currently experimental. In accordance with consensus reports from the European Federation of Periodontology and the American Academy of Periodontology, available evidence is largely limited to preclinical studies and early-phase clinical investigations, with insufficient standardized protocols and long-term human data to support routine clinical application [4]. Recent reviews on periodontal 3D bioprinting further emphasize that these technologies remain translational and should be confined to controlled research settings until predictable clinical outcomes are established. Standardized metrics (e.g., digital area analysis) will refine comparisons. In essence, embrasure maintenance demands etiological diagnosis, phenotype consideration, and modality selection [4,28]. Conservative approaches suffice initially, while surgical expertise resolves severity. Integration across disciplines optimizes outcomes by aligning function with aesthetics.

## Conclusions

Embrasure space maintenance represents a cornerstone of periodontal therapeutics, integrating anatomical preservation with targeted reconstruction to sustain aesthetic and functional integrity. The interdental papilla, shaped by bone proximity, biotype, and contact dynamics, serves as both barrier and aesthetic element, vulnerable to inflammatory, traumatic, and developmental insults. Pathophysiological insights reveal progressive tissue collapse, necessitating early intervention to avert black triangles and their sequelae. Non-surgical strategies, hyaluronic acid volumization, platelet-rich biologics, orthodontic closure, and restorative masking, offer accessible, low-morbidity options for mild-to-moderate defects, achieving modest yet clinically meaningful gains with patient-centered appeal. Surgical grafting, via advanced flap configurations, delivers superior durability for severe loss, restoring height and contour through biological augmentation. Technique selection hinges on defect anatomy, vascular constraints, and operator skill, with microsurgery enhancing predictability. Multidisciplinary collaboration emerges as pivotal, harmonizing orthodontics, prosthetics, and periodontology for holistic resolution. Maintenance regimens, emphasizing atraumatic hygiene and surveillance, safeguard gains. Emerging biomaterials and digital tools herald enhanced precision and regeneration. Integration of biologic modifiers with microsurgical grafting and orthodontic refinement currently offers the most predictable pathway to complete and stable papillary reconstruction. Ultimately, successful embrasure management transcends technique, rooted in etiological correction and individualized planning. By prioritizing prevention and evidence-based progression from conservative to invasive modalities, clinicians can mitigate the global impact of papillary loss, fostering resilient periodontal health and patient satisfaction.
